# High Incidence of Diabetes after Stroke in Young Adults and Risk of Recurrent Vascular Events: The FUTURE Study

**DOI:** 10.1371/journal.pone.0087171

**Published:** 2014-01-23

**Authors:** Loes C. A. Rutten-Jacobs, Pim A. J. Keurlings, Renate M. Arntz, Noortje A. M. Maaijwee, Henny C. Schoonderwaldt, Lucille D. Dorresteijn, Maureen J. van der Vlugt, Ewoud J. van Dijk, Frank-Erik de Leeuw

**Affiliations:** 1 Department of Neurology, Radboud University Medical Center, Donders Institute for Brain, Cognition and Behaviour, Nijmegen, The Netherlands; 2 Department of Internal Medicine, Canisius Wilhelmina Hospital, Nijmegen, The Netherlands; 3 Department of Neurology, Medisch Spectrum Twente, Enschede, The Netherlands; 4 Department of Cardiology, Radboud University Medical Center, Nijmegen, The Netherlands; Maastricht University Medical Center, Netherlands

## Abstract

**Background:**

Diabetes diagnosed prior to stroke in young adults is strongly associated with recurrent vascular events. The relevance of impaired fasting glucose (IFG) and incidence of diabetes after young stroke is unknown. We investigated the long-term incidence of diabetes after young stroke and evaluated the association of diabetes and impaired fasting glucose with recurrent vascular events.

**Methods:**

This study was part of the FUTURE study. All consecutive patients between January 1, 1980, and November 1, 2010 with TIA or ischemic stroke, aged 18–50, were recruited. A follow-up assessment was performed in survivors between November 1, 2009 and January 1, 2012 and included an evaluation for diabetes, fasting venous plasma glucose and recurrent vascular events. The association of diabetes and IFG with recurrent vascular events was assessed by logistic regression analysis, adjusted for age, sex and follow-up duration.

**Results:**

427 survivors without a medical history of diabetes were included in the present analysis (mean follow-up of 10.1 (SD 8.4) years; age 40.3 (SD 7.9) years). The incidence rate of diabetes was 7.9 per 1000 person-years and the prevalence of IFG was 21.1%. Patients with diabetes and IFG were more likely to have experienced any vascular event than those with normal fasting glucose values (OR 3.5 (95%CI 1.5–8.4) for diabetes and OR 2.5 (95%CI 1.3–4.8) for IFG).

**Conclusions:**

Diabetes or IFG in young stroke survivors is frequent and is associated with recurrent vascular events. Regular screening for IFG and diabetes in this population, yields potential for secondary prevention.

## Introduction

Patients, who suffered a stroke at young age, are at high risk of recurrent vascular events and death [1]–[3]. Because of the young age of these patients, the initial stroke as well as possible recurrent vascular events have a large impact on number of years lost to ill-health, disability and early death. Previous studies reported that vascular risk factors are common in these young adults [4], [5]. Secondary prevention measures targeting these vascular risk factors may diminish the risk of recurrent vascular events. However, risk factors that emerge *after* a young stroke often may go undetected in many patients as current protocols and guidelines only recommend screening of young stroke patients in the acute phase and only few months thereafter [6].

Risk of recurrent vascular events seems especially high in young stroke patients with a medical history of diabetes [7]. In both the general population and in stroke patients over 65 years, also impaired fasting blood glucose (IFG) or impaired glucose tolerance, conditions that precede diabetes, have been associated with an increased risk of vascular events [8], [9]. Moreover, more than half of older stroke patients, who were not previously known to have diabetes, was diagnosed to have either impaired glucose tolerance or diabetes three months after stroke [10]. Analogous to these older stroke patients, young stroke patients without a medical history of diabetes at the time of their index event may still develop IFG or incident diabetes after their young stroke as well. Particularly since regular monitoring of glucose levels after the acute phase of stroke in young adults without diabetes is seldom performed. Glucose control in patients with IFG or incident diabetes could be an important way to reduce risk of recurrent vascular events [11]. However, the incidence of diabetes and IFG after stroke in young adults is currently unknown. Moreover, we are not aware of any study that investigates the association between impaired fasting blood glucose and recurrent vascular events in young stroke patients.

Therefore, we first investigated the incidence of diabetes after a mean follow-up of 10 years in survivors of a young TIA or ischemic stroke. Secondly, we investigated whether impaired fasting blood glucose and diabetes at follow-up were associated with the occurrence of vascular events during follow-up.

## Methods

### Patients and study design

This study is a part of the “***F***ollow-***U***p of ***T***ransient ischemic attack and stroke patients and ***U***nelucidated ***R***isk factor ***E***valuation” (***FUTURE***) study, a prospective cohort study of prognosis of stroke in young adults [2], [12]_ENREF_2.The Medical Review Ethics Committee region Arnhem-Nijmegen approved the study.

In short, the FUTURE study comprised all consecutive patients aged 18 through 50 years with a TIA, ischemic stroke or intracerebral hemorrhage admitted to the Radboud university medical center from January 1, 1980 until November 1, 2010. Only patients with TIA or ischemic stroke without a medical history of diabetes, who survived until the follow-up assessment, were included in the present study. Exclusion criteria were cerebral venous sinus thrombosis and retinal infarct.

To minimize bias resulting from changing diagnostic techniques, the World Health Organization definitions for TIA and stroke were used [13], [14]. The definition of TIA included a rapidly evolving focal neurologic deficit, without positive phenomena such as twitches, jerks or myoclonus, with vascular cause only and persisting for a period of less than 24 hours. Stroke was defined as focal neurologic deficit persisting for more than 24 hours. Stroke was subdivided into ischemic and hemorrhagic stroke, on the basis of radiological findings.

Patients were identified through a prospective registry of all patients with young stroke that has been maintained at our centre, beginning in 1978 [15], with a standardized data collection of baseline and clinical characteristics, including demographic data, stroke subtype and vascular risk factors [12]. Assessment of both the etiology (Trial of Org 10172 in Acute Stroke Treatment [TOAST] classification) [16] and severity (National Institutes of Health Stroke Scale [NIHSS]) [17] was performed retrospectively in all cases on the basis of medical records, because these scales did not exist when a substantial number of our patients experienced their index event. In comparison to the original TOAST classification [18], the presently used classification has an additional category, “likely large-artery atherosclerosis” [16]. Atherothrombotic stroke is defined as patients with (1) an ipsilateral internal carotid stenosis >50% (in NASCET criteria), or (2) an ipsilateral stenosis >50% of another intra/extracranial artery, or (3) mobile thrombus in the aortic arch. Likely atherothrombotic stroke is defined as patients with no evidence of atherothrombotic stroke with (1) an ipsilateral internal carotid stenosis <50%, or (2) an ipsilateral stenosis <50% of another intra/extracranial artery, or (3) aortic arch plaques >4 mm in thickness without a mobile component, or (4) a history of myocardial infarction or coronary revascularization, (5) a history of documented peripheral arterial disease, or (6) at least two risk factors for atherosclerotic disease: arterial hypertension (treated or known blood pressure before stroke >135/85 mm Hg or hypertensive retinopathy), diabetes mellitus (treated or known blood fasting glucose >7 mmol/dl), current smoking (or smoking stopped within the last 6 months), high cholesterol (treated or known low-density lipoprotein before the stroke >160 mg/dl).

Patients alive were invited for follow-up assessment between November 1, 2009 and January 1, 2012. Participants provided written informed consent.

### Diabetes and impaired fasting glucose

To answer the first research question, the incidence of diabetes was the primary outcome measure, either diagnosed during follow-up or at the follow-up assessment.

The detection of incident diabetes during follow-up was done by a two step approach. First patients were asked whether diabetes was diagnosed during the follow-up period, by means of a standardized structured questionnaire. If so, patients' general practitioner was contacted to verify the diagnosis systematically, and to ascertain information about the plasma glucose level, type of diagnosed diabetes and initiated treatment.

Secondly, venous plasma samples were taken from all participants at the follow-up assessment after overnight fasting to measure plasma glucose. Whenever glucose was ≥5.6 mmol/L, the patient was sent to the general practitioner to obtain a second fasting venous plasma glucose.

Incident diabetes was defined as: 1) treatment with antidiabetic medication or a diagnosis of diabetes (confirmed by a physician) during the follow-up period or 2) two consecutive fasting venous plasma glucose levels of ≥7.0 mmol/L at the follow-up assessment.

Regarding the second research question, secondary outcomes were the prevalence of diabetes or IFG and the occurrence of vascular events in relation to fasting blood glucose levels at the follow-up assessment. IFG was only assessed at the follow-up assessment, defined as a fasting blood glucose of 5.6 mmol/L–6.9 mmol/L.

### Vascular events

Patients were evaluated for recurrent vascular events by means of a standardized, structured questionnaire [3]. Whenever a recurrent event was suspected, information retrieved was verified and adjudicated by physicians from the appropriate specialty (FEdL, EvD, MvdV).

A composite vascular event was defined as the combination of stroke (ischemic or hemorrhagic), myocardial infarction, and cardiovascular procedures (coronary artery bypass grafting, percutaneous transluminal coronary angioplasty, carotid endarterectomy or other peripheral arterial revascularization procedures), whichever occurred first. Separate analyses were done for stroke and other arterial events.

### Statistical analysis

To answer the first research question, the incidence rate of diabetes was calculated for stroke subtypes. To answer the second research question, fasting blood glucose values at the follow-up assessment were categorized into normal fasting blood glucose (<5.6 mmol/L), impaired fasting blood glucose (5.6 mmol/L–6.9 mmol/L) and diabetes (≥7.0 mmol/L or incident diabetes during follow-up). Baseline characteristics were compared between patients without diabetes or impaired fasting glucose and patients with diabetes or impaired fasting glucose using Student's t test, Mann-Whitney U test or chi-square-test whenever appropriate. Odds ratios were calculated for the association between fasting blood glucose categories at the follow-up assessment and the occurrence during follow-up of the composite vascular event, other arterial events and stroke separately, adjusted for age of the index stroke, sex, and follow-up duration.

Analyses were done using IBM SPSS Statistics version 20. Two-sided P values of less than 0.05 were considered to indicate statistical significance.

## Results

**Figure 1:**
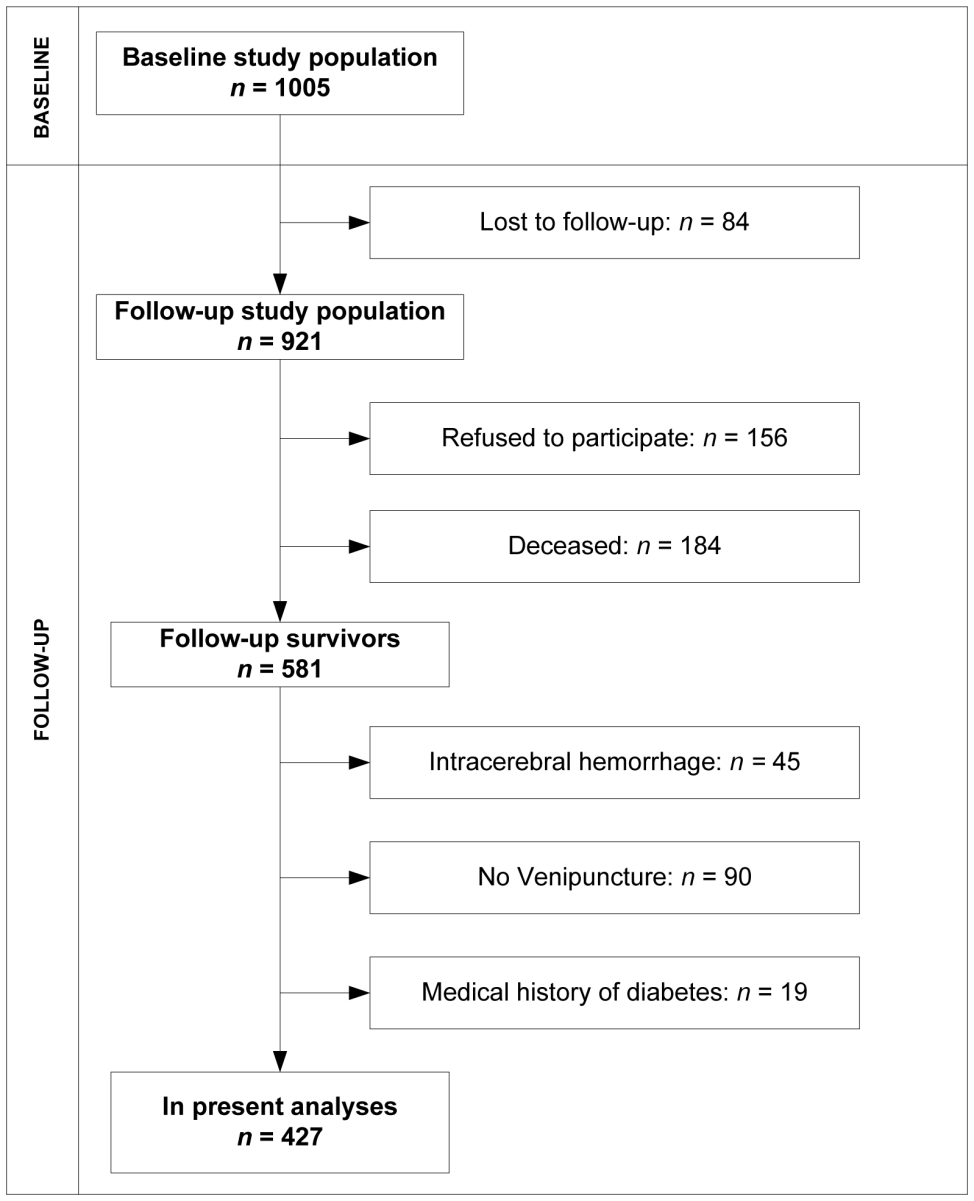
Flowchart of the study population.

427 patients completed follow-up assessment (Figure 1). Baseline characteristics are presented in table 1. There were no differences in baseline characteristics between participants and non-participants (patients lost to follow-up, patients with no venipuncture or patients who refused), except for history of TIA (3.5% in participants and 0.7% in nonparticipants).

**Table 1:** Baseline characteristics of patients.

	Total	TIA	Ischemic stroke
n (% of total)	427 (100)	156 (36.5)	271 (63.5)
Mean age at event, years (SD)	40.3 (7.9)	41.3 (7.8)	39.9 (7.8)
Male	190 (44.5)	71 (45.5)	119 (43.9)
Median NIHSS at admission (IQR)*	2 (0–6)	0 (0–1)	4 (2–8)
Mean follow-up, years (SD)	10.1 (8.3)	8.9 (8.5)	10.9 (8.2)
TOAST			
Atherothrombotic stroke	33 (7.7)	9 (5.8)	24 (8.9)
Likely atherothrombotic stroke	61 (14.3)	27 (17.3)	34 (12.5)
Cardioembolic stroke	44 (10.3)	15 (9.6)	29 (10.7)
Small vessel occlusion	41 (9.6)	7 (4.5)	34 (12.5)
Rare causes	66 (15.5)	16 (10.3)	50 (18.5)
Multiple causes	10 (2.3)	3 (1.9)	7 (2.6)
Unknown cause	172 (40.3)	79 (50.6)	93 (34.3)
Risk factors in medical history			
Previous TIA	15 (3.5)	8 (5.1)	7 (2.6)
Previous stroke	6 (1.4)	2 (1.3)	4 (1.5)
Hypertension	101 (23.7)	46 (29.5)	55 (20.3)
Atrial fibrillation	6 (1.4)	2 (1.3)	4 (1.5)
Smoking†	196 (46.8)	55 (35.9)	141 (53.0)
Excess alcohol consumption‡	27 (6.3)	11 (7.1)	16 (5.9)
Family history of diabetes§	175 (41.4)	69 (45.1)	106 (39.3)

Abbreviations: TIA, transient ischemic attack; SD, standard deviation; NIHSS, National Institute of Health Stroke Scale; IQR, interquartile range; TOAST, Trial of Org 10172 in Acute Stroke Treatment.

Data are given as number (percentage) or otherwise stated

* Scores range from 0 to 42 with higher scores on the scale indicating worse stroke severity. 0.5% of NIHSS was missing.

† Smoking was defined as smoking at least 1 cigarette a day in the year prior to the event. 1.9% of data on smoking was missing.

‡ Excess alcohol consumption was defined as consuming more than 200 grams of pure alcohol per week

§ First degree family member. 0.9% of data on family history of diabetes was missing.

After a mean follow-up of 10.1 years (SD 8.4), diabetes was diagnosed in 11 TIA patients (7.1%) and 23 ischemic stroke patients (8.5%), resulting in an incidence rate per 1000 person years of 7.9 and 7.8 respectively. Among those without diabetes at follow-up, 83 patients (21.1%) had an IFG (5.6–6.9 mmol/L) and 310 patients (78.9%) had normal blood glucose values.

Compared with patients without IFG or incident diabetes at the follow-up assessment, patients with incident diabetes were at baseline more often older, had a longer mean follow-up duration, had a likely atherothrombotic stroke, a medical history of hypertension, a medical history of smoking and a family history of diabetes (Table 2). Compared with patients without IFG or incident diabetes at the follow-up assessment, patients with IFG at the follow-up assessment were at baseline more frequently men, had a higher age, a longer mean follow-up duration, a likely atherothrombotic stroke and a medical history of hypertension.

**Table 2:** Presence of baseline factors in patients with incident diabetes or impaired fasting glucose at follow-up.

	No diabetes or IFG	Diabetes		*p* *	IFG	*p* †		
n (% of total)	310 (72.6)	34 (8.0)			83 (19.4)			
Mean age at event, years (SD)	39.2 (8.2)	44.5 (4.5)	0.002		42.8 (6.5)		0.001	
Male	123 (39.7)	16 (47.1)	0.41		51 (61.4)		<0.001	
Median NIHSS at admission (IQR)‡	2 (0–6)	2 (1–4)	0.82		3 (0–6)		0.31	
Mean follow-up, years (SD)	8.7 (7.8)	16.7 (8.0)	<0.001		12.6 (8.6)		0.001	
TOAST								
Atherothrombotic stroke	20 (6.5)	5 (14.7)	0.08		8 (9.6)		0.32	
Likely atherothrombotic stroke	30 (9.7)	14 (41.2)	<0.001		17 (20.5)		0.007	
Cardioembolic stroke	35 (11.3)	1 (2.9)	0.15		8 (9.6)		0.67	
Small vessel occlusion	34 (11.0)	0	0.06		7 (8.4)		0.50	
Rare causes	56 (18.1)	1 (2.9)	0.03		9 (10.8)		0.14	
Multiple causes	7 (2.3)	1 (2.9)	1.00		2 (2.4)		1.00	
Unknown cause	128 (41.3)	12 (35.3)	0.50		32 (38.6)		0.65	
Risk factors in medical history								
Previous TIA	10 (3.2)	1 (2.9)	1.00		4 (4.8)		0.51	
Previous stroke	5 (1.6)	1 (2.9)	1.00		0		0.37	
Hypertension	58 (18.7)	14 (41.2)	0.002		29 (34.9)		0.002	
Smoking§	133 (43.6)	23 (67.6)	0.008		40 (50.0)		0.31	
Excess alcohol consumption||	19 (6.1)	3 (8.8)	0.71		5 (6.0)		0.97	
Family history of diabetes¶	124 (40.5)	20 (58.8)	0.04		31 (37.3)		0.60	

Abbreviations: IFG, impaired fasting glucose; TIA, transient ischemic attack; SD, standard deviation; NIHSS, National Institute of Health Stroke Scale; IQR, interquartile range; TOAST, Trial of Org 10172 in Acute Stroke Treatment.

Data are given as number (percentage) or otherwise stated

* *p* values refer to a comparison between patients with incident diabetes and patients with no IFG or diabetes

† *p* values refer to a comparison between patients with IFG and patients with no IFG or diabetes

‡ Scores range from 0 to 42 with higher scores on the scale indicating worse stroke severity. 0.4% of NIHSS was missing.

§ Smoking was defined as smoking at least 1 cigarette a day in the year prior to the event. 2.9% of data on smoking was missing.

|| Excess alcohol consumption was defined as consuming more than 200 grams of pure alcohol per week

¶ First degree family member. 1.0% of data on family history of diabetes was missing.

At follow-up, 12 patients with incident diabetes (35.3%) had experienced any vascular event (composite event) and 7 patients (20.6%) of them experienced more than one event; 4 patients (11.8%) had at least one stroke and 10 patients (29.4%) had experienced at least one other arterial event. Among patients with IFG at follow-up, 21 patients (25.3%) had experienced any vascular event and 6 patients (7.2%) of them experienced more than one event; 10 patients (12.9%) had at least one stroke and 11 patients (13.3%) had experienced at least one other arterial event. Among patients with normal fasting blood glucose levels at follow-up, 30 patients (9.7%) had experienced any vascular event and 6 patients (1.9%) of them; 24 patients (7.7%) had experienced at least one stroke and 8 patients (2.6%) had experienced at least one other arterial event. In all three fasting blood glucose groups, the proportion of patients on antiplatelet medication at discharge did not differ between patients who experienced a recurrent vascular event compared with patients who did not experience a recurrent vascular event during follow-up.

After adjusting for age of index stroke, sex and follow-up duration, patients with diabetes and IFG were more likely to have experienced any vascular event during follow-up than those with normal fasting blood glucose values (OR 3.5 (95%CI 1.5–8.4) for diabetes and OR 2.5 (95%CI 1.3–4.8) for IFG). Risk for the recurrence of stroke was not different for patients with incident diabetes and IFG compared with those with normal fasting blood glucose values (OR 1.2 (95%CI 0.4–4.0) for diabetes and OR 1.4 (95%CI 0.6–3.3) for IFG). Risk of other arterial events was increased in patients with diabetes and IFG compared with those with normal fasting blood glucose levels (OR 8.4 (95% CI 2.7–26.4) for diabetes and (OR 3.6 (95%CI 1.3–9.6) for IFG).

## Discussion

We demonstrated that 8% of young stroke survivors developed diabetes during a mean follow-up of 10 years after stroke, which is more than two times higher than expected compared with persons from a Dutch general practitioner registry with similar age and sex [19]. Moreover, we showed that among those patients without diabetes at the follow-up assessment, 21% had impaired fasting blood glucose values. In our study, both patients with diabetes and patients with IFG at the follow-up assessment were about three times more likely to experience any vascular event during follow-up than those with normal fasting blood glucose values.

To our knowledge, our study is the first to evaluate the incidence of diabetes after stroke in young adults and to study the association between fasting blood glucose values and recurrent vascular events. Moreover, our study has the longest follow-up period reported and one of the largest study populations in the field of young stroke. Collecting data all in one site allowed us to collect baseline and follow-up information according to identical procedures in all patients thereby reducing the risk of information bias.

Our study has some limitations. First, it may be that not all cases of young stroke in our catchment area were included in our cohort, because our cohort is a single-center, hospital-based study, rather than community-based. Only those patients who sustained a fatal stroke, who were not admitted to our hospital, would not have been included in our study. Patients who survive usually visit a university medical center during the course of their disease. In addition, there are no restrictions to be admitted to our hospital and we included all consecutive cases admitted. We therefore presume that our study population is a representative sample of Dutch patients with young stroke, although formal data are lacking to prove this generalizability.

Second, we investigated the association of IFG and diabetes with recurrent vascular events during follow-up in a cross-sectional analysis, on average 10 years after the index event in patients that survived until the follow-up assessment.

Thus the measurement of blood glucose values is done after a recurrent event occurred. This may have induced survivor bias. IFG and diabetes may be associated with the severity of the recurrent event and as a consequence, patients with IFG and diabetes may be underrepresented in survivors with recurrent events, which may have attenuated the association between IFG/diabetes and recurrent events.

Furthermore, IFG was only measured at the follow-up assessment, whereas for diabetes also a diagnosis established during the follow-up period was taken in account. Diabetes that developed during follow-up might otherwise have been missed at the follow-up assessment due to initiated treatment.

Third, some patients were lost to follow-up or refused to participate, which potentially could have resulted in selection bias. However, non-participants did not differ in baseline characteristic from participants, making selection bias in this group unlikely.

Fourth, our study has a long inclusion period, during which diagnostic equipment, acute treatment and secondary prevention have improved. However, this is an unavoidable feature of a long-term follow-up study. Furthermore, the long follow-up period might have resulted in recall bias with respect to vascular events. However, this probably would have underestimated the association between diabetes and recurrent vascular events, since the incidence of diabetes was strongly related to the number of follow-up years.

Fifth, secondary prevention might have influenced our results. In our study about 90% of all patients used secondary preventive medication at discharge. Consequently the shown risk of recurrent vascular events might be an underestimation attributable to the use of this preventive medication. Sixth, as is reflected by the wide CIs, estimates for some subgroups that contain only a few patients might be unstable and should therefore be interpreted with caution.

So far, the only studies reporting on epidemiology of diabetes in young stroke patients restricted their reports to diabetes diagnosed prior to stroke. The proportion of patients with a medical history of diabetes varied widely in these studies, ranging from 2–12% [7], [20], [21]. Our observed prevalence of diabetes based on the medical history of 4.9% is in the middle of this range.

We showed in univariate analysis that incident diabetes after TIA or ischemic stroke was associated with age, likely atherothrombotic stroke and family history of diabetes, which are among well established risk factors for diabetes in the general population. In addition, we showed that both patients with diabetes and patients with IFG were far more likely to have experienced any arterial event during follow-up than those with normal fasting blood glucose values. These results suggest an intimate relationship in young stroke patients between pre-existent vulnerability to atherosclerosis and incident diabetes, which is an atherogenic risk factor itself. However, it is also possible that diabetes was already present but not revealed during the index event.

Incident diabetes or IFG was not associated with recurrent stroke. An explanation might be that diabetes needs to be present for many years to be a risk factor for recurrent stroke. This is in line with a previous study in young adults with ischemic stroke that showed that among patients with type 1 diabetes, duration of diabetes was on average 10 years longer in those with recurrent stroke versus those without recurrent stroke [7]. Another explanation for the lack of association might be the possibility of index event bias [22]. In a study investigating recurrence, patients are included based on the occurrence of the first event that is similar to the recurrent event. This has an effect on the distribution of risk factors in this selected population and the association of these risk factors with the outcome of interest.

The high incidence of diabetes during our long follow-up period, but also the high proportion of patients with IFG, emphasizes that young stroke survivors remain vulnerable to the development of (risk factors for) vascular disease, even decades after their initial stroke. Active screening for IFG and diabetes after stroke in young adults may allow for early diagnoses of IFG and diabetes and thereby provide a therapeutic window to lower the risk of recurrent vascular events. Similar to the general population, young stroke patients with a higher age, having other vascular risk factors or a family history of diabetes, might benefit the most from active screening.

To conclude, IFG and diabetes after stroke in young patients may remain unnoticed in many patients. A regular screening for IFG and diabetes after young stroke, particularly in those with increasing age, having other vascular risk factors or a family history of diabetes, yields potential for secondary prevention.
